# Photobiomodulation as a contemporary antioxidant intervention in myocardial infarction

**DOI:** 10.1113/EP093042

**Published:** 2026-10-30

**Authors:** Gabriel Matheus Da Silva Batista, Ighor Luiz Azevedo Teixeira, Ednei Luiz Antonio, Andrey Jorge Serra

**Affiliations:** ^1^ Programa de Pós‐graduação em Cardiologia Universidade Federal de São Paulo São Paulo Brazil

**Keywords:** heart failure, myocardial infarction, photobiomodulation

## Abstract

Myocardial infarction (MI) triggers a complex cascade of structural and functional alterations that frequently culminate in adverse cardiac remodelling and heart failure. Central to this process is the excessive production of reactive oxygen species (ROS), which disrupt mitochondrial function, impair calcium handling, and activate pro‐apoptotic and pro‐fibrotic pathways. Photobiomodulation (PBM) has emerged as a non‐invasive therapeutic approach capable of modulating several key biological processes involved in post‐infarction remodelling. Preclinical studies consistently demonstrate that PBM reduces infarct size, attenuates ventricular dilation, and improves both systolic and diastolic function. Through the absorption of red to near‐infrared light by mitochondrial chromophores, particularly cytochrome *c* oxidase, PBM enhances cellular bioenergetics, modulates ROS production and activates redox‐sensitive signalling pathways. These effects promote a shift from detrimental oxidative stress toward controlled ROS signalling and, in turn, facilitate cytoprotective, anti‐inflammatory and pro‐angiogenic responses. In addition, PBM promotes the mobilization of progenitor cells, contributing to tissue repair and microvascular restoration. Although still limited, the available clinical evidence suggests that PBM may confer modest improvements in cardiac function. However, variability in treatment protocols, delivery methods and study designs currently precludes definitive conclusions regarding its clinical efficacy. In summary, PBM represents a promising strategy for modulating oxidative stress after MI, integrating metabolic, redox and signalling pathways to attenuate cardiac remodelling. Further well‐designed clinical studies are required to establish standardized protocols and to clarify its therapeutic potential in cardiovascular medicine.

## Abstract (highlights)

- **What is the topic of this review?**
  This topic of this review is photobiomodulation, which has emerged as a non‐invasive therapeutic approach capable of modulating several key biological processes involved in post‐infarction cardiac remodelling.
- **What advances does it highlight?**
  Photobiomodulation attenuates post‐infarction cardiac remodelling by inducing redox hormesis. It represents a promising, non‐invasive adjunctive strategy for mitigating myocardial injury and preventing adverse cardiac remodelling after myocardial infarction.

## 1 INTRODUCTION

Myocardial infarction (MI) is considered one of the major threats to human health worldwide, contributing substantially to morbidity and mortality (Salari et al., 2023; Udell et al., 2025). As the disease progresses, the heart may undergo structural remodelling through a complex, multifactorial process that leads to myocardial hypertrophy and, ultimately, heart failure (HF) (Marcos‐Garces et al., 2025). Given that the complete cure rate of MI remains low and functional recovery is often poor, a growing number of studies have investigated targeted pharmacological therapies, exercise training, cell‐based approaches and implantable devices aimed at reducing infarct size and attenuating adverse cardiac remodelling. Nevertheless, potential adverse effects and substantial barriers to clinical translation continue to limit the widespread adoption of these approaches. Thus, there is an urgent need for novel therapeutic strategies capable of improving post‐MI remodelling and, consequently, clinical outcomes.

A growing body of evidence from experimental studies and preliminary clinical trials indicates that photobiomodulation (PBM) is a promising therapeutic approach capable of modulating multiple biological processes involved in post‐MI remodelling (Carlos et al., 2016; Gao et al., 2021). PBM is a non‐invasive technique that uses low‐level light sources, typically delivered as low‐level laser therapy (LLLT) or via light‐emitting diodes (LEDs), to modulate cellular processes. Absorption of red and/or near‐infrared (NIR) light by specific cellular components, mainly within mitochondria, triggers a series of bioenergetic and signalling responses that enhance cellular function and support tissue repair and regeneration (Ganipineni et al., 2023). Accordingly, the cardioprotective action of PBM has been attributed to increased ATP production, release of pro‐angiogenic factors, inhibition of inflammation, and antioxidant effects (Carlos et al., 2016).

An antioxidant role for PBM has also been postulated and may be particularly relevant because prolonged ischaemia promotes excessive production of reactive oxygen species (ROS) (Villa et al., 2025). Excessive ROS production leads to mitochondrial dysfunction, swelling and membrane rupture, ultimately activating programmed cell death pathways (Heusch et al., 2023). Moreover, ROS accumulation is heightened in HF and can activate multiple cellular responses, including cardiomyocyte hypertrophy, cell death and interstitial fibrosis, in part through the activation of matrix metalloproteinases. Excessive ROS production also leads to abnormalities in Ca^2^^+^ cycling and Na^+^/Ca^2^^+^ exchanger activity, which may impair cardiomyocyte electrophysiological function (Jiang et al., 2023; Li et al., 2015; Sawyer, 2011; Wagner et al., 2003).

In this review, we summarize the current state of knowledge on the effects of PBM therapy in MI, with a particular focus on its antioxidant potential to enhance cardioprotection. We highlight key preclinical findings that may support the translation of PBM into effective clinical therapies and discuss the antioxidant mechanisms that may contribute to improvements in disease pathophysiology. Finally, we present the available clinical evidence and discuss its main implications in light of current findings, considering the potential for future application of PBM in clinical practice.

### 1.1 Literature search strategy

This narrative review was based on a comprehensive search of the PubMed, Web of Science and Google Scholar databases. Search terms included ‘photobiomodulation’, ‘low‐level laser therapy’, ‘myocardial infarction’, ‘oxidative stress’ and ‘cardiac remodelling’. We prioritized experimental and clinical studies focused on the antioxidant mechanisms of PBM in the context of ischaemic heart disease. Studies were selected based on their relevance to mitochondrial function, redox signalling and functional cardiac outcomes. To ensure methodological quality and clinical relevance, the preparation of this manuscript followed the Scale for the Assessment of Narrative Review Articles (SANRA), addressing key items such as description of the literature search, scientific reasoning and appropriate presentation of endpoint data (Baethge et al., 2019); the correspondence between each included study and the SANRA items is shown in Table 1.

**TABLE 1:** Mapping of included primary studies to SANRA quality domains.

References	Item 3: Description of the literature search	Item 4: Referencing	Item 5: Scientific reasoning	Item 6: Appropriate presentation of data
Yaakobi et al. (2001)	Preclinical rat model of LLLT (804 nm) via transthoracic route	Peer‐reviewed, fully indexed landmark study on cardiac laser irradiation	Role of heat shock proteins (HSP70 upregulation) in post‐MI cytoprotection	Quantitative structural endpoints (infarct size and ventricular dilation)
Zhang et al. (2010)	Preclinical rat model evaluating red LLLT (635 nm) via direct cardiac route	Highly indexed journal in molecular and cellular cardiology	Provides early‐to‐mid phase mechanistic evidence on baseline antioxidant enzyme responses (SOD and MDA)	Complete echocardiographic indices (LVDd, LVSd, EF, LVFS) and biochemical assays
Yang et al. (2013)	Preclinical rat model evaluating red LLLT (635 nm) via direct cardiac route	Rigorously peer‐reviewed cardiovascular laser therapy reference	Explores the limits of antioxidant enzyme consumption and collagen content (fibrosis) in cardiac remodelling	Reports structural (infarct size, wall thickness) and localized redox markers (SOD, MDA)
Biasibetti et al. (2014)	Preclinical rat model of PBM (660 nm) via peripheral skeletal muscle	High‐quality indexed reference in laser medicine and systemic effects	Contributes to scientific reasoning regarding the systemic and peripheral antioxidant action of PBM	Peripheral muscular oxidative stress markers (SOD, GPx, DCFH) and cardiac haemodynamic endpoints
Malinovskaya et al. (2008)	Preclinical rat model comparing He–Ne laser vs. wideband red light	Peer‐reviewed experimental biology and medicine reference	Compares coherent vs. non‐coherent light sources and their impact on acute lipid peroxidation (MDA)	Specific acute biochemical outcomes (MDA, SOD) post‐reperfusion
Manchini et al. (2014)	Preclinical rat model evaluating LLLT (660 nm) via direct cardiac route	Open‐access, peer‐reviewed, extensively cited reference	Identifies the central nitric oxide pathway balance via eNOS upregulation and iNOS downregulation	Structural (infarct size), pulmonary congestion, echocardiographic (FAC, E/A ratio) and biochemical (plasma NOx) endpoints
Grandinetti et al. (2019)	Preclinical rat model of combined therapy (LLLT 830 nm + Carvedilol) via transthoracic route	Highly cited, peer‐reviewed study in multidisciplinary sciences	Underpins the synergistic effects of pharmacological and optical therapies, highlighting lipid peroxidation control (4‐HNE)	Structural (LVDA, LVSA), functional (FAC, LVEDP) and oxidative stress (4‐HNE, CP, SOD, CAT) parameters
Feliciano et al. (2021)	Preclinical rat model of LLLT (660 nm) via direct cardiac application	State‐of‐the‐art peer‐reviewed molecular cardiology study	Delivers critical insights into transcriptional and post‐transcriptional pathways of endogenous enzymes	Focuses on high‐quality molecular biology endpoints (GPx, SOD, CAT and HSP70)
Antonio et al. (2025)	Preclinical rat model comparing multi‐dose fluences (357, 714, 1428 J/cm^2^)	Newest peer‐reviewed, high‐density experimental dataset	Formulates the core scientific reasoning for redox hormesis and dose–response biphasic effects	Evaluation of cardiac function (+d*P*/d*t*, −d*P*/d*t*, LVEDP), pulmonary congestion, and oxidative damage biomarkers (4‐HNE, CP, SOD, CAT, HSP70)

*Note*: SANRA items 1 (justification of the article's importance for the readership) and 2 (statement of concrete aims or formulation of questions) apply to the review as a whole and are addressed in the ‘Introduction’.

Abbreviations: +d*P*/d*t*, left ventricular maximum rate of pressure rise; −d*P*/d*t*, left ventricular maximum rate of pressure decline; 4‐HNE, 4‐hydroxynonenal; CAT, catalase; CP, carbonylated protein; DCFH, 2′,7′‐dichlorodihydrofluorescein oxidation; E/A ratio, ratio between early (E) and late (A) diastolic filling velocities; EF, left ventricular ejection fraction; eNOS, endothelial nitric oxide synthase; FAC, left ventricular fractional area change; GPx, glutathione peroxidase; He–Ne, helium–neon; HSP70, heat shock protein 70; iNOS, inducible nitric oxide synthase; LLLT, low‐level laser therapy; LVDA, left ventricular diastolic area; LVDd, left ventricular end‐diastolic diameter; LVEDP, left ventricular end‐diastolic pressure; LVFS, left ventricular fractional shortening; LVSA, left ventricular systolic area; LVSd, left ventricular end‐systolic diameter; MDA, malondialdehyde; MI, myocardial infarction; NOx, nitrite/nitrate concentration; PBM, photobiomodulation; SANRA, Scale for the Assessment of Narrative Review Articles; SOD, superoxide dismutase.

## 2 EFFECTS OF PBM ON POST‐INFARCTION CARDIAC REMODELLING

Most of the evidence supporting the cardioprotective effects of PBM comes from experimental studies conducted in rats (Table 1). LLLT has emerged as the predominant approach, applied with distinct fluence and irradiance parameters, either directly to the myocardium or via a transthoracic route. Studies have also varied the timing of PBM application after MI, and there is limited evidence regarding the impact of prolonged treatment extending into the progression toward overt HF.

Reducing infarct size can attenuate cardiac dilation, improve ventricular function and delay the onset of HF (Udell et al., 2025). In this context, although findings across studies are not entirely consistent, PBM appears to be a promising strategy, as several studies reported that LLLT reduces infarct size, although others found no significant effect (Table 2). Oron et al. (2001) were among the first to apply LLLT directly to the heart in infarcted dogs and rats. They reported a 6.5% reduction in mortality and smaller infarct size after 5 weeks of follow‐up. Consistent with this, we previously demonstrated that direct cardiac application of LLLT reduced both infarct size and the incidence of large infarcts during the early phase of post‐infarction cardiac remodelling (Manchini et al., 2014).

**TABLE 2:** Photobiomodulation therapy parameters and repercussions in infarcted myocardium.

	Photobiomodulation protocol							Cardiac impact		
References	Species	Model	Fluence/irradiance (J/cm^2^)	Wavelength (nm)	Route	Frequency application	Structural	Systolic performance	Diastolic performance	Oxidative stress markers
Yaakobi et al. (2001)	Rat	LLLT	0.27 (post‐MI); 0.81 (3 days post‐MI)	804	Transthoracic	2× (10–15 min and 3 days post‐MI)	MI size: ↓ LVD: ↓			HSP70: ↑
Zhang et al. (2010)	Rat	LLLT	0.96	635	Heart	1× (3 weeks post‐MI)	LVDd: ↔ LVSd: ↔	EF: ↔ LVFS: ↔		SOD: ↑ MDA: ↓
Yang et al. (2013)	Rat	LLLT	0.96	635	Heart	1× (3 weeks post‐MI)	MI size: ↓ CC: ↓ LVWT: ↑			SOD: ↓ MDA: ↑
Biasibetti et al. (2014)	Rat	LLLT	3 and 21	660	Gastrocnemius	10× (daily, 4 weeks post‐MI)	MI size: ↔	LVSP: ↔	LVEDP: ↔	SOD: ↓ (gastrocnemius) GPx: ↔ DCFH: ↔
Malinovskaya et al. (2008)	Rat	He–Ne laser/Wideband red light	3	640 (bandwidth of 70 nm)	Sinoatrial node	1× (post‐reperfusion)				MDA: ↓ SOD: ↓ (He‐Ne laser) SOD: ↑ (wideband red light)
Grandinetti et al. (2019)	Rat	LLLT	714	830	Transthoracic	3× a week for 4 weeks	MI size: ↔ LVDA: ↓ LVSA: ↔ PC: ↓ MM/BW: ↔	FAC: ↑ LVSP: ↔ +d*P*/d*t*: ↔	LVEDP: ↓ ‐dP/dt: ↔	4‐HNE: ↓ CP: ↔ SOD: ↔ CAT: ↔
Feliciano et al. (2021)	Rat	LLLT	1.15	660	Heart	1× post‐MI				GPx: ↔ SOD: ↔ CAT: ↔ HSP70: ↔
Manchini et al. (2014)	Rat	LLLT	22.5	660	Heart	1× post‐MI	MI size: ↓ MM/BW: ↔ PC: ↔ LVDA: ↔ LVSA: ↓	FAC: ↑	E wave: ↔ A wave: ↔ E/A ratio: ↓	iNOS: ↓ eNOS: ↑ Plasma NOx: ↑
Antonio et al. (2025)	Rat	LLLT	357, 714 and 1428	830	Heart/transthoracic	3× a week for 4 weeks	MI size: ↔ PC: ↓ (357 and 714 J/cm^2^) LVDA: ↔ LVSA: ↔ CC: ↓ NV: ↓ (357 J/cm^2^)	LVSP: ↑ FAC: ↔ +d*P*/d*t*: ↑	LVEDP: ↓ (357 and 714 J/cm^2^) −d*P*/d*t*: ↑ (357 and 714 J/cm^2^) E wave: ↔ A wave: ↔ E/A ratio: ↔	4‐HNE: ↓ CP: ↓ (357 and 714 J/cm^2^) SOD: ↑ CAT: ↑ (714 J/cm^2^) HSP70: ↔

Abbreviations: BW, body weight; CAT, catalase; CC, collagen content; CP, carbonylated protein; DCFH, 2′,7′‐dihydrodichlorofluorescein oxidation; EF, left ventricular ejection fraction; eNOS: endothelial nitric oxide synthase; E/A ratio, relation between velocity of E and A waves; FAC, left ventricle fractional area change; GPx, glutathione peroxidase; HSP70, heat shock protein 70; iNOS, inducible nitric oxide synthase; LLLT, low‐level laser therapy; LVDA, left ventricular diastolic area; LVDd: left ventricular end‐diastolic diameter; LVEDP, left ventricular end‐diastolic pressure; LVFS: left ventricular fractional shortening; LVSA, left ventricular systolic area; LVSd: left ventricular end‐systolic diameter; LVWT, left ventricular wall thickness; LVSP, left ventricular systolic pressure; MDA, malondialdehyde; MI, myocardial infarction; MM, myocardial mass; NOx, nitrite/nitrate concentration; PC, pulmonary congestion; SOD, superoxide dismutase; +d*P*/d*t*, left ventricular maximum change in pressure; −d*P*/d*t*, left ventricular minimum change in pressure; 4‐HNE, 4‐hydroxynonenal; ↑ increase; ↓ decrease; ↔ no significant difference.

Cavitary dilation is a common post‐infarction outcome, referred to as eccentric hypertrophy (Ha et al., 2021). This adaptive mechanism may transiently help maintain cardiac performance through the Frank–Starling mechanism. However, its long‐term compensatory capacity is limited, and progressive cardiac dysfunction and HF often develop (Zornoff et al., 2009). We and other investigators have demonstrated that LLLT has a beneficial role by attenuating left ventricular (LV) dilation (Grandinetti et al., 2019; Manchini et al., 2014; Yaakobi et al., 2001). These structural effects may partly account for the improvements in LV systolic function reported in some studies (Grandinetti et al., 2019; Manchini et al., 2014). Considering that the LV mass‐to‐volume ratio decreases in eccentrically hypertrophied hearts, and in light of Laplace's law, it is conceivable that a higher LV fractional area change and +d*P*/d*t* (Grandinetti et al., 2019; Manchini et al., 2014) may reflect a less dilated ventricular cavity, despite reduced intrinsic myocardial contractile capacity (Serra et al., 2010). The potential of PBM to attenuate diastolic dysfunction is also encouraging. In the early phase of the disease, we showed that infarcted rats treated with LLLT exhibited improved LV filling patterns on echocardiographic assessment (Manchini et al., 2014). In subsequent studies, LLLT also improved diastolic function in rats treated for up to 4 weeks after MI (Antonio et al., 2025; Grandinetti et al., 2019).

Anti‐inflammatory and pro‐angiogenic effects are commonly regarded as important mediators of the cardioprotective effects of LLLT (Carlos et al., 2016; Grandinetti et al., 2019; Tuby et al., 2006). More recently, we showed that LLLT temporally modulates the gene expression of several extracellular matrix components (Feliciano et al., 2022) and attenuates myocardial fibrosis in female infarcted rats (Antonio et al., 2025). A single study has also shown that LLLT increases the expression of proteins involved in calcium transient regulation, suggesting that PBM may modulate both myocardial contractility and relaxation (Antonio et al., 2025). Finally, modulation of oxidative stress represents a plausible mechanism underlying the effects of PBM in the infarcted myocardium. After MI, marked ROS production may occur, promoting lipid peroxidation, protein oxidation, mitochondrial dysfunction and activation of pro‐apoptotic pathways, all of which contribute to adverse cardiac remodelling.

By interacting with cytochrome *c* oxidase (CCO) in the mitochondrial respiratory chain and with calcium ion channels (possibly through light absorption by opsins), PBM may modulate ATP production and ROS generation and increase nitric oxide (NO) availability (Hamblin, 2017). This may activate redox‐sensitive signalling pathways, thereby upregulating antioxidant defences and reducing oxidative stress. Collectively, these mechanisms could reduce cell death, limit infarct expansion and improve cardiac performance (Carlos et al., 2016). Taken together, these findings position modulation of oxidative stress as a central mechanistic axis underlying the cardioprotective effects of PBM after MI (Fig. 1).

**FIGURE 1:**
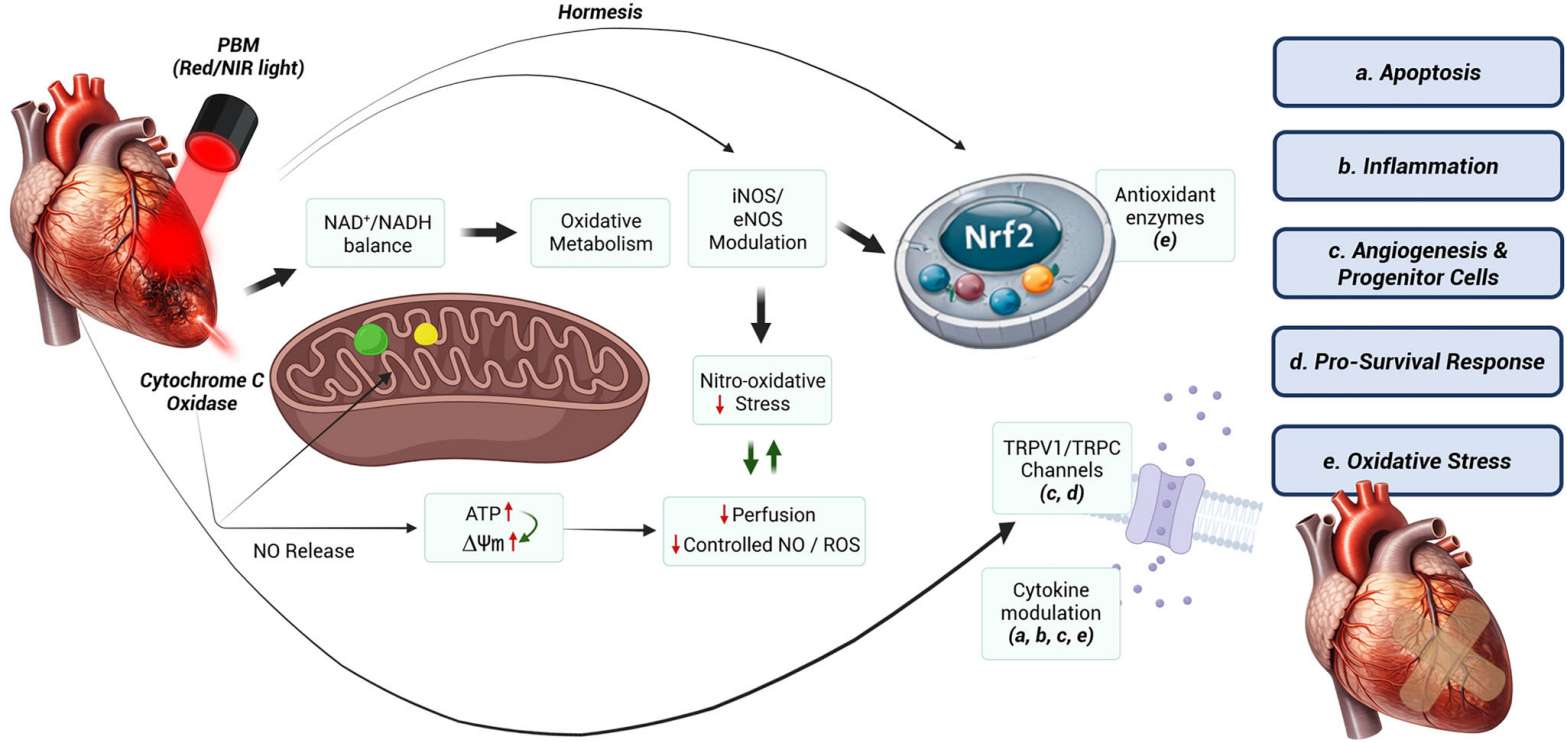
Overview of the antioxidant and cardioprotective actions of photobiomodulation in myocardial infarction, including cytochrome *c* oxidase activation, nitric oxide (NO) signalling, and Nrf2‐dependent redox regulation. The right hand quadrants illustrate the final phenotypic outcomes governed by the pathways modulated by photobiomodulation, with each quadrant identified by the letters *a–e*. ATP, adenosine triphosphate; eNOS, endothelial nitric oxide synthase; iNOS: inducible nitric oxide synthase; Nrf2, nuclear factor erythroid 2‐related factor 2; ROS, reactive oxygen species; TRPC, transient receptor potential canonical; TRPV1, transient receptor potential cation channel subfamily V member 1.

## 3 INSIGHTS INTO THE ANTIOXIDANT EFFECTS OF PBM

### 3.1 Primary photochemical reactions

PBM occurs when photons within the therapeutic window (600–1300 nm) are absorbed by intracellular chromophores. CCO, also known as complex IV of the mitochondrial respiratory chain, is considered the main mitochondrial photoreceptor for PBM and represents the terminal enzyme of the electron transport chain (Karu, 2000, 2010).

The interaction between PBM and CCO is primarily determined by the spectral properties of the metal cofactors present within the complex. The haem groups and copper centres of CCO display relevant absorption bands in the visible red (∼620–680 nm) and near‐infrared (∼760–830 nm) regions, allowing photons within these spectral ranges to be absorbed directly by the enzyme. In particular, haem *a* and haem *a*_3_ exhibit absorption bands in the red and NIR regions, making them plausible intracellular chromophores responsible for the initial transduction of the photonic stimulus (Karu & Kolyakov, 2005). Photon absorption may modulate the redox state of these cofactors and transiently influence electron transfer within CCO, thereby promoting increased oxygen consumption, higher mitochondrial membrane potential and enhanced ATP production (Wong‐Riley et al., 2005).

In addition, an interaction between PBM and NO reversibly bound to the catalytic haem *a*_3_–CuB centre of CCO has been proposed. Under conditions of metabolic stress or injury, such as hypoxia or myocardial ischaemia, NO may compete with molecular oxygen for binding to the haem *a*_3_ site, thereby partially inhibiting enzymatic activity, reducing electron flow through the respiratory chain and decreasing ATP production (Shiva & Darley‐Usmar, 2003). Notably, upon absorption of red or NIR photons by CCO chromophores, PBM may modulate the so‐called ‘nitric oxide paradox’. Under these conditions, inhibitory NO is photodissociated from the haem *a*_3_ centre, restoring oxygen binding and the catalytic activity of the complex (Karu, 2010). As a result, electron flux is enhanced, mitochondrial membrane potential (ΔΨ_m_) is re‐established and oxidative phosphorylation is upregulated (Hamblin, 2017).

### 3.2 Mitochondrial bioenergetic recovery

During myocardial ischaemia, NADH accumulates, shifting the NAD^+^/NADH redox balance and reflecting impaired mitochondrial respiration and substrate oxidation. Reactivation of CCO by PBM, together with the associated increase in electron transport, drives the oxidation of NADH to NAD^+^, contributing to the restoration of mitochondrial redox homeostasis (Gonçalves de Faria et al., 2021). This process is critical for the resumption of oxidative metabolism, since several TCA cycle dehydrogenases depend on NAD^+^ availability to maintain metabolic flux. Thus, modulation of respiratory activity by PBM may facilitate the recovery of aerobic metabolism in ischaemic cardiomyocytes, thereby contributing to the restoration of ATP production and to the functional recovery of cardiac tissue (Hamblin, 2017).

It has been shown that inducible NO synthase (iNOS) mRNA expression is increased after MI in rats, contributing to enhanced nitrosative and oxidative stress (Feng et al., 2001; Wang et al., 1999). Conversely, the expression of endothelial NO synthase (eNOS), which constitutively produces NO and supports vascular homeostasis through vasodilatory and antithrombotic effects, is downregulated in the weeks following MI (Bauersachs et al., 1999). PBM attenuates the increase in iNOS mRNA levels and restores eNOS mRNA expression at this time point, compared with untreated animals (Manchini et al., 2014). These findings suggest that PBM may reduce oxidative and nitrosative stress, mitochondrial damage and cell death associated with iNOS upregulation while simultaneously promoting local vasodilation and improving tissue perfusion through restoration of eNOS activity (Kashiwagi et al., 2023).

In addition, PBM stimulates NO production through non‐enzymatic pathways. Light exposure enhances the nitrite reductase activity of myoglobin, particularly in the presence of sodium nitrite, promoting local NO generation even when NOS activity is impaired (Kashiwagi et al., 2023). The current hypothesis is that inhibitory NO previously bound to CCO becomes dissociated, thereby restoring electron transport chain activity and increasing ΔΨ_m_ (Ravera et al., 2019). This process may increase ATP synthesis and modulate ROS production. Mitochondria‐derived ROS act as signalling mediators, activating redox‐sensitive pathways that regulate cellular survival, antioxidant defence and tissue regeneration (DiGiovanni et al., 2025; Karu et al., 2005).

### 3.3 ROS‐mediated signalling and oxidative eustress

The mitochondrial bioenergetic recovery induced by PBM triggers a secondary signalling phase characterized by controlled redox modulation. Reactivation of the mitochondrial electron transport chain, driven by increased CCO activity, restores oxidative phosphorylation and elevates ATP production (Wong‐Riley et al., 2005; Zhang et al., 2021). Enhanced mitochondrial respiration is accompanied by a transient increase in electron leakage from complexes I and III, resulting in the brief generation of low levels of ROS, including superoxide and hydrogen peroxide.

The transient increases in mitochondrial ROS induced by PBM reflect a hormetic dose–response relationship, consistent with the Arndt–Schulz principle, in which low levels of oxidative stress promote adaptive signalling, whereas excessive stimulation shifts the redox balance toward oxidative distress (Huang et al., 2011). In contrast to the pathological oxidative stress observed during MI, particularly after ischaemia–reperfusion injury, oxidative eustress represents a regulated redox state in which ROS act as intracellular second messengers. In cardiomyocytes exposed to PBM (850 nm, 1 J/cm^2^), mitochondrial ROS oxidize specific cysteine residues in redox‐sensitive proteins, thereby regulating signalling molecules, kinases and transcription factors involved in cellular adaptation and survival (Bahr et al., 2024).

A central mechanism is redox hormesis, in which mild oxidative stimuli trigger compensatory antioxidant responses that enhance cellular resilience to further oxidative challenges; nuclear factor erythroid 2‐related factor 2 (Nrf2) is a key mediator of this response. Under basal conditions, Nrf2 is retained in the cytoplasm through its interaction with Kelch‐like ECH‐associated protein 1 (Keap1), which targets it for ubiquitination and proteasomal degradation. Oxidation of critical cysteine residues within Keap1 disrupts this interaction, resulting in Nrf2 stabilization and nuclear translocation. In the nucleus, Nrf2 binds to antioxidant response elements (AREs) and activates the transcription of cytoprotective and antioxidant genes. Nrf2 forms heterodimers with small Maf (sMaf) proteins and recruits coactivators such as CREB‐binding protein/p300 and promotes chromatin remodelling through histone acetylation, enhancing transcriptional accessibility and RNA polymerase II recruitment (Calabrese & Kozumbo, 2021; Chen & Maltagliati, 2018; Wang et al., 2020).

Key target genes include antioxidant and detoxifying enzymes such as superoxide dismutase, catalase, glutathione peroxidase, haem oxygenase‐1 and NAD(P)H:quinone oxidoreductase 1, as well as aldehyde‐metabolizing enzymes such as aldehyde dehydrogenase 2. Collectively, these effectors enhance redox buffering capacity, promote glutathione‐dependent conjugation and facilitate the oxidation of reactive aldehydes, including 4‐hydroxynonenal, thereby limiting damage induced by lipid peroxidation. Activation of the Nrf2‐dependent antioxidant network represents a central mechanism through which PBM attenuates oxidative injury during MI, promoting cardiomyocyte survival and enhancing cellular resilience to subsequent stress (Lu et al., 2022; Wang et al., 2020)

### 3.4 Calcium and kinase signalling pathways

Emerging evidence suggests that PBM exerts part of its molecular effects through modulation of light‐ and thermosensitive ion channels. Specifically, NIR laser irradiation (940 nm, 3 J/cm^2^) has been shown to activate the transient receptor potential vanilloid 1 (TRPV1) channel in human buccal fat pad adipose stem cells (Gholami et al., 2022; Ohba et al., 2007). Furthermore, PBM (830 nm, 3 J/cm^2^) has been shown to downregulate transient receptor potential canonical‐6 (TRPC‐6) in muscle from mdx mice, suggesting a potential mechanism for modulating calcium handling. Although these channels are intrinsically involved in cardiac remodelling and fibrosis (Covatti et al., 2023), their regulation by PBM in the infarcted myocardium has not yet been investigated. Although extrapolation from non‐cardiac models should be interpreted with caution, these findings provide a plausible mechanistic framework by which PBM may modulate cytosolic Ca^2^^+^ homeostasis. The resulting transient increase in intracellular Ca^2^^+^ may act as a critical second messenger, coupling photonic stimulation to mitochondrial bioenergetics and redox signalling through controlled ROS generation in cardiomyocytes (Falcón et al., 2020; Numaga‐Tomita et al., 2019; Popoiu et al., 2023).

The cytosolic Ca^2^^+^ signal stimulates mitochondrial activity and promotes controlled ROS production. At non‐lethal levels, ROS act as redox signalling molecules, reversibly modifying signalling proteins. The Ca^2^^+^–ROS axis converges on nuclear factor‐κB (NF‐κB) activation, potentially linking PBM therapy to the regulation of inflammatory and survival genes. Whereas a transient ROS burst activates NF‐κB to promote the expression of cell survival genes, chronic pathological activation of NF‐κB is attenuated by PBM (Cheng et al., 2023; Chu et al., 2018; Lee et al., 2016). In the context of ischaemia, PBM stabilizes hypoxia‐inducible factor‐1α (HIF‐1α), which together with activation of the Akt pathway – as demonstrated in a murine model of myocardial injury (Jiang et al., 2026) – stimulates expression of vascular endothelial growth factor. This response promotes angiogenesis and contributes to the restoration of microcirculation in the infarct border zone (Huang et al., 2021; Li et al., 2020).

### 3.5 Tissue repair and systemic responses

PBM drives coordinated reparative responses in the ischaemic myocardium by regulating mitochondrial ROS production and limiting excessive oxidative damage during reperfusion (Hamblin & Liebert, 2022). Attenuation of oxidative stress can suppress pro‐apoptotic signalling pathways, thereby reducing cardiomyocyte loss in the infarct border zone. In parallel, improved mitochondrial function and Akt‐dependent signalling support endothelial cell survival and stimulate angiogenic processes, facilitating microvascular remodelling and enhancing tissue perfusion (Liao et al., 2022).

PBM also modulates the oxidative–inflammatory axis that characterizes the post‐MI milieu, decreasing the expression of pro‐inflammatory cytokines such as interleukin (IL)‐1β (IL‐1β), IL‐6 and tumour necrosis factor‐α, while promoting anti‐inflammatory mediators such as IL‐10. This shift toward a pro‐resolving immune environment limits secondary oxidative injury and facilitates the transition from inflammation to tissue repair (Grandinetti et al., 2019; Hentschke et al., 2013).

Emerging evidence indicates that PBM promotes the mobilization of bone marrow‐derived progenitor cells into the circulation, a process that appears to be mediated by ROS‐dependent signalling pathways and by chemokine gradients generated in the injured tissue. These circulating cells are recruited to the ischaemic myocardium, where they contribute to paracrine signalling, angiogenesis and tissue remodelling (Mansano et al., 2023). In this context, adipose‐derived stem cells exhibit enhanced survival and regenerative activity under PBM stimulation, indicating that modulation of oxidative stress is a key mechanism linking mitochondrial photoreception to both local cardioprotection and systemic regenerative responses after MI (Batista et al., 2025; Chaves Dos Santos et al., 2025; de Souza Vieira et al., 2026).

Taken together, these findings indicate that PBM acts as a regulator of post‐MI oxidative stress, shifting the cellular redox balance from detrimental oxidative injury toward controlled ROS signalling that promotes cardiomyocyte survival and regenerative processes.

## 4 CLINICAL DATA AND RECOMMENDATIONS FOR FUTURE RESEARCH

Preclinical studies are encouraging and support the hypothesis that patients with MI may benefit from PBM therapy. Improvements in cardiac function may be associated with modulation of inflammation, angiogenesis and mitochondrial function, and with attenuation of oxidative stress. However, clinical data remain limited and are derived mainly from non‐randomized trials with small and heterogeneous samples.

In the interventional setting, an early strategy was to deliver PBM within the myocardial reperfusion window. Two randomized studies reported that intracoronary LLLT resulted in smaller late lumen loss in patients undergoing percutaneous coronary intervention (PCI) compared with non‐irradiated controls. These findings were associated with a lower restenosis rate, attenuated expression of transforming growth factor‐β1 and fibroblast growth factor‐2, and higher nitrite/nitrate concentrations (Derkacz et al., 2014, 2017). In a subsequent study, although LV ejection fraction (LVEF) was not improved, significantly lower troponin‐T levels were observed when LLLT was applied to the tibial bone prior to PCI (Elbaz‐Greener et al., 2018). In contrast, studies applying LLLT in patients undergoing coronary artery bypass grafting have not demonstrated beneficial effects on markers of myocardial injury or oxidative stress (Moghadam, 2019; Stein et al., 2018). Regarding the potential benefits of PBM in HF, one study reported a 30% increase in LVEF and a 26% increase in serum NO levels in adult patients with chronic systolic HF (Sayed et al., 2023). In contrast, another study in 10 post‐MI patients reported no improvement after 15 sessions of LLLT (Razzaghi et al., 2024).

Despite the promising benefits of PBM, several translational barriers still need to be addressed. A primary challenge is the dosing uncertainty associated with the hormetic response (Arndt–Schulz principle). Preclinical evidence shows that, whereas low doses promote adaptive signalling, excessive irradiation may lead to oxidative distress. For example, our recent experimental study showed that low and intermediate fluences (357 and 714 J/cm^2^) improved several indices of post‐infarction HF, whereas a higher fluence (1428 J/cm^2^) produced less consistent effects (Antonio et al., 2025). In addition, tissue penetration represents a major hurdle in humans. Unlike small rodent models, in which light is applied directly to the heart or transthoracically; in human, photons must traverse skin, bone and muscle, resulting in substantial attenuation before reaching the myocardium. Thus, the incident optical dose does not necessarily reflect the dose actually received by cardiomyocytes. Although direct measurement of the intracellular PBM dose is currently not feasible in vivo, computational approaches such as Monte Carlo simulations may provide estimates of myocardial light delivery.

Furthermore, differences between preclinical animal models and human pathology limit clinical translation. Preclinical studies typically use young, healthy animals with induced MI, whereas human patients are frequently older and present with comorbidities (e.g., diabetes, hypertension) that substantially alter baseline redox signalling and the inflammatory environment. These factors, combined with the lack of parameter standardization and the heterogeneity of delivery routes, contribute to the inconsistent clinical results reported to date. Therefore, adequately powered randomized controlled trials are required to determine whether the preliminary effects of PBM translate into sustained clinical benefit or, instead, represent transient responses dependent on specific experimental conditions.

## AUTHOR CONTRIBUTIONS

Gabriel Matheus Da Silva Batista, Ighor Luiz Azevedo Teixeira and Ednei Luiz Antonio conceived or designed the work. Andrey Jorge Serra drafted the work or revised it critically for important intellectual content. All authors have read and approved the final version of this manuscript and agree to be accountable for all aspects of the work in ensuring that questions related to the accuracy or integrity of any part of the work are appropriately investigated and resolved. All persons designated as authors qualify for authorship, and all those who qualify for authorship are listed.

## CONFLICT OF INTEREST

None declared.

## GENERATIVE AI STATEMENT

The GPT Plus website has been used to scan the manuscript for spelling and grammatical errors.
